# Assessment of Individual-Level Exposure to Airborne Particulate Matter during Periods of Atmospheric Thermal Inversion

**DOI:** 10.3390/s22197116

**Published:** 2022-09-20

**Authors:** Rok Novak, Johanna Amalia Robinson, Tjaša Kanduč, Dimosthenis Sarigiannis, David Kocman

**Affiliations:** 1 Department of Environmental Sciences, Jožef Stefan Institute, 1000 Ljubljana, Slovenia; 2 Jožef Stefan International Postgraduate School, 1000 Ljubljana, Slovenia; 3 Center for Research and Development, Slovenian Institute for Adult Education, Ulica Ambrožiča Novljana 5, 1000 Ljubljana, Slovenia; 4 Environmental Engineering Laboratory, Department of Chemical Engineering, Aristotle University of Thessaloniki, 54124 Thessaloniki, Greece; 5 HERACLES Research Centre on the Exposome and Health, Center for Interdisciplinary Research and Innovation, 54124 Thessaloniki, Greece; 6 Department of Science, Technology and Society, University School of Advanced Study IUSS, 27100 Pavia, Italy

**Keywords:** personal exposure, particulate matter, atmospheric thermal inversion, personal monitoring, exposure assessment

## Abstract

Air pollution exposure is harmful to human health and reducing it at the level of an individual requires measurements and assessments that capture the spatiotemporal variability of different microenvironments and the influence of specific activities. In this paper, activity-specific and general indoor and outdoor exposure during and after a period of high concentrations of particulate matter (PM), e.g., an atmospheric thermal inversion (ATI) in the Ljubljana subalpine basin, Slovenia, was assessed. To this end, personal particulate matter monitors (PPM) were used, worn by participants of the H2020 ICARUS sampling campaigns in spring 2019 who also recorded their hourly activities. ATI period(s) were determined based on data collected from two meteorological stations managed by the Slovenian Environmental Agency (SEA). Results showed that indoor and outdoor exposure to PM was significantly higher during the ATI period, and that the difference between mean indoor and outdoor exposure to PM was much higher during the ATI period (23.0 µg/m^3^) than after (6.5 µg/m^3^). Indoor activities generally were associated with smaller differences, with cooking and cleaning even having higher values in the post-ATI period. On the other hand, all outdoor activities had higher PM values during the ATI than after, with larger differences, mostly >30.0 µg/m^3^. Overall, this work demonstrated that an individual-level approach can provide better spatiotemporal resolution and evaluate the relative importance of specific high-exposure events, and in this way provide an ancillary tool for exposure assessments.

## 1. Introduction

Airborne particulate matter (PM) negatively impacts human health, reduces life expectancy, and increases mortality, and is a particularly important health risk in urban environments as traffic and other factors additionally contribute to higher concentrations of PM and other pollutants [1,2,3,4,5].

A common approach to assessing exposure is using monitoring stations that measure outdoor concentration levels of various pollutants and require compliance with regulatory protocols, which makes them the reference standard in an urban environment for evaluating long-term trends, outdoor concentrations, and city-wide exposure assessments [6]. On the other hand, they are expensive to operate, physically large, and consequently limited in number and coverage. While there are several options to use data from monitoring stations to estimate indoor exposure [7], static outdoor stations are not able to capture the variability of exposure based on an individual’s activities and daily movement trajectory [8]. Multiple studies have shown that collecting data on air quality and exposure on an individual level, in contrast to city-wide monitoring, provides higher spatiotemporal granularity to observe individual-level exposure and daily fluctuations in diverse indoor and outdoor urban settings, including the impact of atmospheric thermal inversions (ATIs) [9,10,11]. Exposure assessments based on individual-level measurements usually show higher recorded values than estimates based on data from monitoring stations [12,13], and therefore assessments that use community average concentrations of PM can underestimate the health burden of air pollution [14]. Personal monitoring devices can be used to estimate negative health outcomes from exposure to higher PM concentrations [15] as well as the importance of socioeconomic variables, e.g., sociodemographic status, urban mobility, and living conditions, when assessing exposure to PM [16]. To further explore the applicability of PM monitors in individual-level exposure research, their performance should be assessed within a period that would show distinct differences in exposure during activities and in microlocations that the individual records. A period with persistent ATIs in a suitable wintertime environment (e.g., an alpine basin) could provide the necessary conditions, as it is characterized by two clearly delimited periods of high and low concentrations of PM.

ATIs that occur in urban environments, as a consequence of atypical temperature gradients, produce a “cap” which reduces the diffusion of dust, smoke, and other air pollutants [17] and can cause concentrations of air pollutants to increase, with a high level of spatiotemporal variability throughout the urban environment [18,19]. An elevated level of exposure during ATIs can lead to detrimental health effects, mostly as an increase in the incidence of acute respiratory diseases, asthma, and cardiovascular diseases [20,21,22]. However, increased exposure on an individual level during ATIs is still poorly understood.

A unique set of conditions present in a subalpine basin (Ljubljana, Slovenia), e.g., concave shape, extended periods of anticyclonic conditions, and drag associated with the complex topography, resulting in frequent foggy days and ATIs, which in turn cause a buildup of PM [23]. The Ljubljana basin experiences frequent short-lived inversions in all seasons, though persistent inversions occur mostly in the colder part of the year [24]. Meteorological conditions are a driving factor in determining air quality in Ljubljana, and can surpass the importance of emission ceilings [25]. Although air quality has been improving in most European cities over the past decade, Ljubljana, as of 27 July 2022, ranks 279 out of 344 cities from the European Environment Agency (EEA) member countries in terms of air quality, with an average PM concentration of 15.7 µg/m^3^, measured in 2020 and 2021, labeled as “poor air quality” by the EEA [26]. ATIs, compounded by the poor air quality in Ljubljana during winter, temporarily increase exposure to PM and offer a distinctive perspective on high-exposure events in urban environments, which individual-level monitoring could help to assess in more detail.

This study used next-generation sensing and monitoring (NGSM) technology—a wearable PM monitor—to determine how these types of devices could provide fine-grained spatiotemporal resolution of personal exposure to PM_10_ in a period of persistent ATIs and immediately after, based on individual activities and microlocations. Individual-level exposure assessments were based on data obtained from personal PM monitors (PPM) used as part of the ICARUS H2020 project [27], where participants carried the devices for one week in a heating and non-heating season, and additionally provided hourly data on their activities, transport mode, and microlocations [28].

## 2. Materials and Methods

### 2.1. Collecting Particulate Matter Data

**Figure 1:**
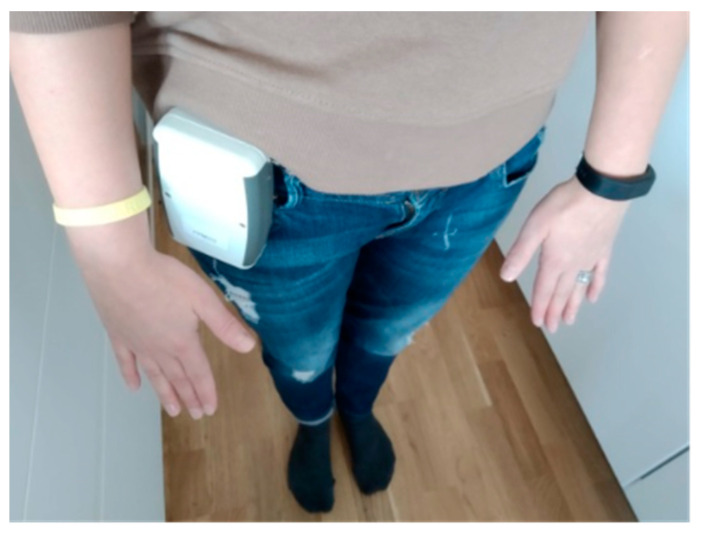
PPM device (white box attached to clothes) worn by a participant.

For assessing exposure on an individual level a wearable device called the PPM (shown in Figure 1) was used, which provided data on PM_1_, PM_2.5_, and PM_10_ concentrations, ambient temperature, relative humidity, and location/GPS data with minute resolution. The devices were designed and constructed for the ICARUS project [29] by IoTech Telecommunications, Thessaloniki, Greece [30] and are based on the Arduino platform and the Plantower, Beijing, China, pms5003 sensor [31,32]. To determine whether the device provided data that was fit for purpose and accurate, a validation was conducted by collocating the PPM with a GRIMM (Durag Group, Hamburg, Germany) model 11-A (1.109) aerosol spectrometer, which showed that the PPM had relatively high accuracy and was fit for purpose, further described in Novak et al. [33].

Participants were instructed to wear the PPM for the entire duration of the study or have it placed near them if they performed sedentary or stationary activities, e.g., office work or sleeping. The data were collected on an internal SD card and exported via a web app/portal and stored on a local drive. Each participant had to fill out a time activity diary (TAD) and indicate what the characteristic activity was that they were performing each hour of the day for seven days. Data were used from the ICARUS heating season sampling campaign, which took place from 16 February 2019 to 12 March 2019.

To observe the trend of PM_10_ concentrations in Ljubljana during and after the period with persistent ATIs, city-wide data on PM_10_ concentrations (30 min values) and meteorological conditions (temperature and wind speed) were provided by the Slovenian Environmental Agency (SEA) [34] from the urban background reference station in the Bežigrad district of Ljubljana. Preliminary observations showed that a period with persistent ATIs could have occurred in Ljubljana in the same period as the heating season sampling campaign. Data from the monitoring stations were collected for the period from 10 February 2019 to 15 March 2019 to provide additional context.

### 2.2. Determining ATIs

ATIs were determined by analyzing temperature gradients between stations at different elevations, per the Largeron and Staquet [35] pseudo-vertical temperature gradient method (TGM), which presupposes two assumptions: (1) horizontal homogeneity of the temperature field and (2) the quasi-linearity of the temperature profile. When these considerations are met, the ratio of the temperature and height difference between the stations (ΔT*/*Δz) can be used to determine the stability of the boundary layer when the inversion occurs [35]. Kikaj et al. [23] determined that these assumptions were met for low- and medium-lying stations in and around the Ljubljana basin in the colder months of the year. High-elevation stations showed moderate correlation coefficients when calculating horizontal air temperature homogeneity and were, in the scope of this paper, only used to estimate the height of the inversion layer by determining if the inversion persisted up to the height of the station.

**Figure 2:**
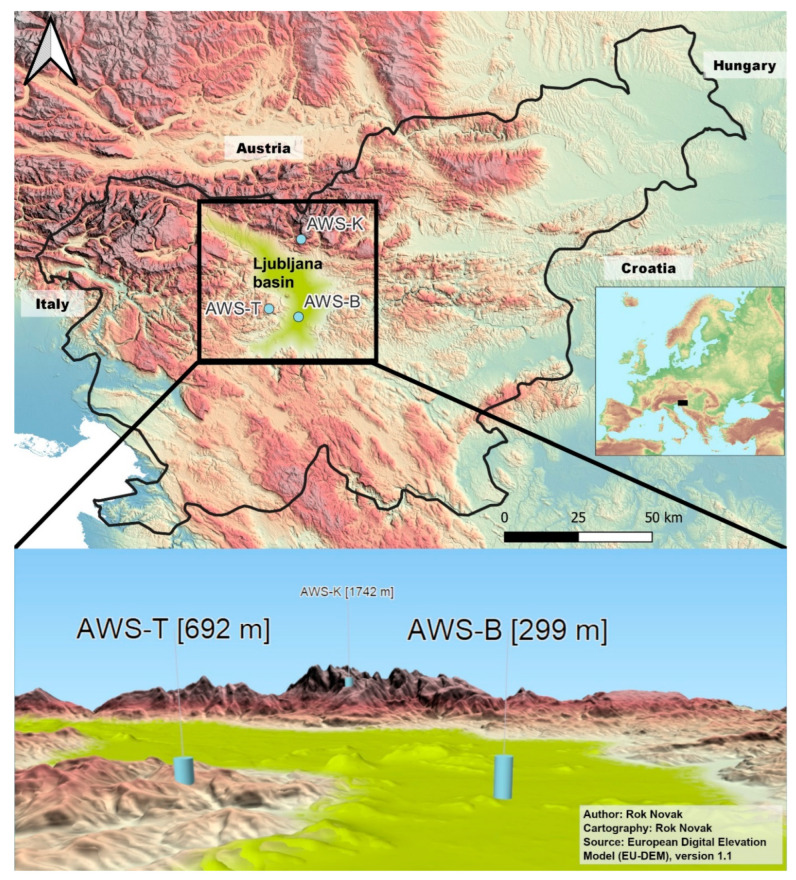
Geographical locations of automatic weather stations Bežigrad, Topol, and Krvavec. Top: Locations on a topographical map of Slovenia and neighboring countries with the location of the Ljubljana basin. Bottom: 3D visualization of locations and their respective elevations with vertical exaggeration (2), designed with the Qgis2threejs plugin [36] in QGIS 3.20.1-Odense [37].

Measurements were collected from three automatic weather stations (AWS), one low-lying station located in the center of Ljubljana (station Bežigrad—AWS-B), one medium-lying station situated on a hill at the border of the basin (Topol—AWS-T) and one high-lying station at the northern border of the basin (Krvavec—AWS-K), shown in Table 1 with their respective elevations, coordinates, and collected parameters. The stations cover the central, western, and northwestern parts of the Ljubljana basin, as shown in Figure 2. All stations measure and report air temperature at 2 m above ground, at 7:00, 14:00, and 21:00, each day.

As per the TGM, the ratio of the temperature difference between stations AWS-B and AWS-T to their difference in elevation (ΔT/Δz) was used to indicate the stability of the boundary layer and consequently when an inversion occurred, as shown in Equation (1):

(1) $$\frac{\Delta T}{\Delta z}=\frac{\Delta T_{T,B}}{\Delta z_{T,B}}\times 10^{3}$$

where $\Delta T_{T,B}$ is the temperature difference between the AWS-T and AWS-B station and $\Delta z_{T,B}$ is the height difference between AWS-T and AWS-B station.

Positive values indicate an ATI, and periods when values consistently show ΔT/Δz > 0 for at least 72 consecutive hours indicate persistent inversions, as shown in Equation (2):

(2) $$\frac{\Delta T}{\Delta z}>0\;\mathrm{for}\;\geq 72\;h$$

The definition of 72 h for a persistent ATI is based on the criteria set by Largeron and Staquet [35].

### 2.3. Treatment of Data from the PPMs and TADs

After harmonizing the data sets of the PPM and TAD (described in detail in Novak et al. [38]), data were selected based on a specific set of criteria: (a) they were part of the heating period data set, (b) data were available for the period when ATIs occurred, (c) the PPM consistently provided data, and (d) the TADs were filled out.

A key procedure was to assign an indoor/outdoor label to each minute value. As the GPS data provided by the PPM did not provide accurate enough spatial resolution to determine if the person was indoors or outdoors, data on activities in the TAD and temperature measured by the PPM were used as follows:

- Outdoor activities in the TAD were: using a bicycle, walking, running outdoors, participating in outdoor sports activities, and three generic labels: “Home.OUT”, “Office.OUT”, and “Other.OUT”. For indoor activities, there were similar generic labels included, “Home.IN”, “Office.IN”, and “Other.IN”, as well as resting and sleeping indoors, playing, indoor sporting activities, cooking, cleaning, and smoking indoors. More specific activities were included in the generic labels;
- Primarily, activity and microlocation labels from the TADs were used to determine if the person was indoors or outdoors. To further refine the accuracy of the indoor/outdoor variable, ambient temperature data recorded by the PPM were used;
- During the observed period from late February to early March in 2019, the outdoor temperatures as measured by AWS-B never exceeded 19 °C in Ljubljana. Using this value as the highest base value gave an approximate highest possible temperature for outdoor activities, though it did have some drawbacks as the device could be exposed to direct sunlight and show higher values than those recorded at automatic weather stations;
- The PPM was collocated with a reference instrument (Testo SE & Co. KGaA, Lenzkirch, Germany, Testo 435-2 sensor with an external IAQ probe [39]) to assess the accuracy of the temperature measurements. Results showed that the PPM had a very high correlation (0.98) with the values recorded by the reference instrument, though the values consistently showed 4.5 °C higher values than the reference instrument. Though the PPM had precise values, they were not accurate. There are several possible causes, most probably due to the positioning of the sensor enclosed in the device close to a warm rechargeable battery. Temperature difference was even higher during the first half hour of charging the battery. This does not affect the outdoor measurements as it is reasonable to assume that the device was not charged during outdoor activities;
- Considering the above (the temperature in Ljubljana never exceeding 19 °C and the 4.5 °C (offset) higher values of the PPM), activities were removed from the outdoor category if they had a temperature above 23.5 °C, and similarly removed from the indoor category if the temperatures were below 23.5 °C.

In some cases, certain inputs in the TADs could overlap between indoor and outdoor microlocations, where a person selected an indoor activity because it represented a majority of the hour, though they spent an amount of time in that same hour outside, e.g., preparing a meal for 40 min then going for a walk would be indicated as an indoor activity for this hour even though the person spent a third of the time outdoors. Using the temperature correction improved the accuracy of the activity dataset.

### 2.4. Data Selection and Evaluation

Exposure was calculated for the period between 16–22 February 2019 and 23 February to 12 March 2019, the first being the period with a persistent ATI event and two days of latency for the PM concentrations as observed at the AWS-B, and the second being the period after the inversion dispersed. Exposure in each respective domain and time period was calculated based on Equation (3):

(3) $$E_{d,p}=\frac{\sum_{i=1}^{n}m_{i}}{n}$$

where *E* is the cumulative exposure, *d* indicates the domain (indoor, outdoor, or activity) and *p* the period (during ATI or post-ATI) of exposure, *m_i_* represents each respective minute measurement in the spatiotemporal period, and *n* the number of measurements made in that period. A cumulative exposure approach was used to determine the baseline differences between the ATI and post-ATI periods, inter-activity differences, and how cumulative exposure assessments based on personal monitors fared in contrast to an assessment based on data collected from monitoring stations. Minute values of PM_10_ collected from the PPMs carried by the participants were aggregated and averaged based on each respective domain (temporal, spatial, activity).

The periods chosen were determined based on the results of the ATI calculations, wind speed data, and data on the height of the inversion layer. Only certain activities from the collected dataset were considered in the scope of this paper; some were removed due to the unavailability of the data in both periods, e.g., smoking and burning of incense/candles. After eliminating participants who didn’t have any PM_10_ data or empty TADs, the period with persistent ATIs had fewer individuals available (3) than the post-ATI period (24). Some of the data were removed from certain participant datasets if they did not meet the required temperature criteria. Mean values with standard deviation were calculated for all indoor and outdoor activities and plotted in boxplots. A one-way ANOVA was performed on the final dataset, in combination with a Tukey’s HSD (honestly significant difference) post-hoc test for pairwise comparisons.

## 3. Results and Discussion

### 3.1. ATIs

**Figure 3:**
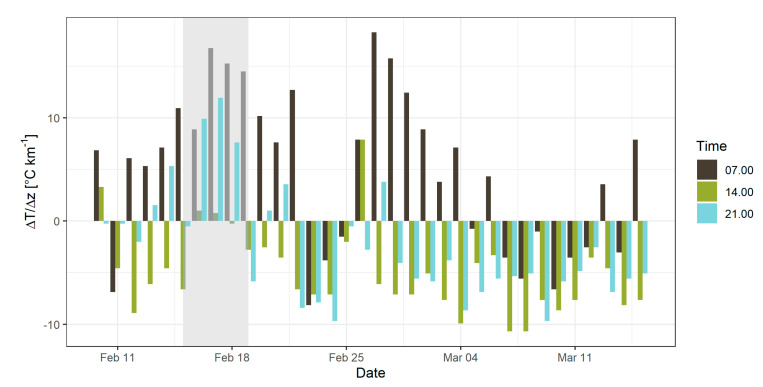
ΔT/Δz between Bežigrad and Topol stations from 10 February to 15 March 2019. Period with persistent ATI shaded in grey.

Figure 3 shows the ΔT*/*Δz values calculated for the period from 10 February to 15 March 2019 to provide some context for the observed ATIs and patterns in the fluctuations of ΔT*/*Δz [°C/km]. An ATI is defined by ΔT*/*Δz > 0 °C/km, which is present several times in Figure 3, most frequently in the mornings (colored black) when the temperatures in the valley were still lower than in higher elevations. This pattern indicates diurnal inversions, formed every night due to radiative cooling of the soil, producing a very stable surface layer particularly associated with alpine valleys [35].

A persistent ATI, defined as ΔT*/*Δz > 0 °C/km for at least 72 consecutive hours, as indicated in Equation (2), is present in only one period, from 16 February to 19 February, shaded gray in Figure 3, with an exception on 18 February at 14:00 with a ΔT*/*Δz of −0.25 °C/km. As this individual observation indicated only a small negative number, the decision was made to include this and the next three measurements in the period of the persistent ATI, as the daily average still showed a high ΔT*/*Δz, as well as the three days following the end of this period. Importantly, the presence of the inversion is key, and the strength of the inversion does not play a vital role in the scope of this research. The days between 20 February and 22 February still experienced frequent inversions and primarily showed high levels of PM_10_ concentrations, and so were consequently included in the post-ATI period to better capture the true exposure associated with ATIs. Persistent ATIs occur in Ljubljana multiple times per year; based on Equation (2) and data collected from SEA [34] there were three periods in 2019 with persistent ATIs: 16 to 19 February, 22 to 26 October, and 23 to 27 December.

The period following the persistent ATI shows sporadic occurrences of inversions during morning and evening measurements, which completely stop on 22 February. After 25 February there is a period of diurnal ATIs in the morning hours with the highest ΔT*/*Δz value on 27 February at 18.3 °C/km. Data from AWS-B also showed that on 23 February average wind speeds increased from 0.2 to 5.0 m/s and average temperatures decreased from 9.1 °C to 1.1 °C.

#### Estimate of Boundary Layer Height

Data collected from the AWS-K station showed that the inversion layer did not surpass the height of the station itself (1742 m) in any of the measurements made at 12:00 in the period between 10 February and 15 March 2019. On the other hand, there were several instances in the 7:00 measuring interval, most common during the period with persistent ATIs. A case of inversion that stood out happened on 17 February 2019 at the 7:00 interval, when the AWS-B station measured a temperature of −2.1 °C, and AWS-K 6.7 °C (a difference of 8.8 °C). Moreover, observing data from the highest-lying station in Slovenia (Kredarica, at elevation 2513 m.a.s.l., some 60 km distance from AWS-B), revealed a temperature of 0.8 °C, indicating that the boundary layer was above this height on 17 February 2019 at 7:00.

### 3.2. PM Measurements at the Monitoring Station

**Figure 4:**
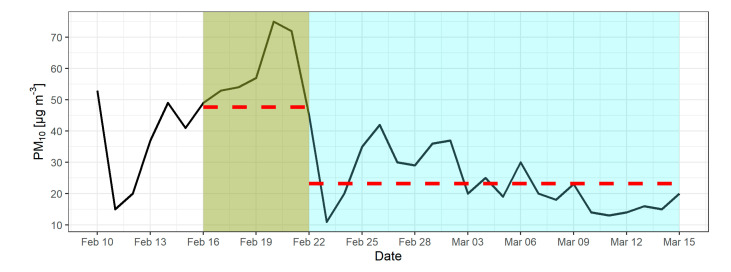
Measured daily PM_10_ concentrations in the observed period from 10 February to 15 March 2019, collected from AWS-B. Persistent ATI period with a latent increase of PM concentrations shaded green, the post-ATI period included in the analysis shaded blue. Dashed red lines show mean PM_10_ values for each period.

As evident in Figure 4, the concentrations of PM_10_ as measured at the AWS-B started increasing as the ATIs became more frequent, peaking on 20 February, a day after the period with persistent ATIs ended. This shows a latency effect of rising PM concentrations in relation to ATIs, as the inversions continued during morning and evening measuring intervals and affected the concentrations of PM_10_. The highest value of PM_10_ was recorded on 20 February, with 75 µg/m^3^, which decreased rapidly and reached its lowest point three days later on 23 February, with 11 µg/m^3^. Mean values for the high-PM period (16–22 February, shaded green in Figure 4) and low-PM period (23 February–15 March, shaded blue in Figure 4) were 47.7 µg/m^3^ and 23.2 µg/m^3^, respectively, shown with dashed lines in Figure 4 for both periods.

### 3.3. Data Collected from PPMs and TADs

The entire ICARUS dataset (in Ljubljana) consisted of 1,439,231 observations of 107 variables, which was refined to 136,115 observations of 32 variables for the purposes of this paper, including timestamps, indoor and outdoor activities, PM_10_ concentrations, and temperature. Next, the data were separated into four groups—indoor and outdoor during the ATI (with 10,622 and 1931 observations, respectively), and indoor and outdoor post-ATI period (with 59,719 and 6664 observations, respectively).

As evident in Table 2 and Table 3, there are certain activities that have a large number of recorded instances, e.g., resting, sleeping, cycling, and generic “home”, “office”, and “other”, and some activities that have few instances, e.g., sports and running. The lowest number of instances (61) are recorded for running during the period with persistent ATIs, which is due to the fact that the recorded temperatures for most of the running period were higher than the elimination criteria for outdoor activities (23.5 °C). Almost all the values for the number of instances per activity in the post-ATI period were higher, as this period was longer and included more participants. Note that the “office” activity in Table 3 shows the number of instances for outdoor activities during the individual’s work hours.

### 3.4. Exposure Assessment

**Figure 5:**
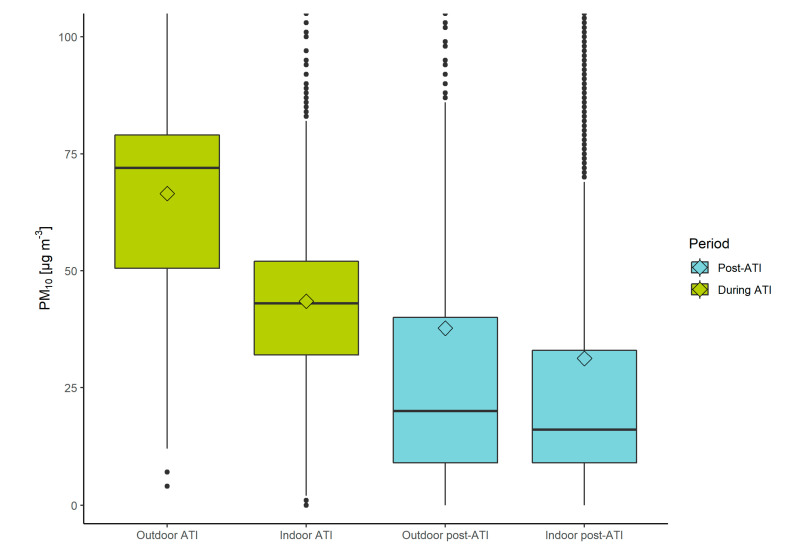
Calculated exposure to PM_10_ indoors and outdoors in the period with a persistent ATI event (in green) and the post-ATI period (blue), collected from the PPM devices. Values above 100 µg/m^3^ were removed from the plot to better visualize the differences between mean (diamond) and median values (line).

Figure 5 shows that exposure to PM_10_ calculated based on Equation (3), as measured by the PPM, was higher indoors and outdoors during the persistent ATI event, compared to the post-ATI period. During the ATI period participants were exposed to a mean PM_10_ concentration of 43.5 µg/m^3^ (σ ± 26.8 µg/m^3^) indoors, and 66.5 µg/m^3^ (σ ± 23.5 µg/m^3^) outdoors, which is in stark contrast with the post-ATI period where indoor and outdoor exposures were 31.2 µg/m^3^ (σ ± 56.8 µg/m^3^) and 37.7 µg/m^3^ (σ ± 96.1 µg/m^3^), respectively. As determined by the ANOVA test (and subsequently the Tukey’s HSD test), the differences between the means were statistically significant, and the results show that there was a real difference between all four microlocation combinations (indoors during and after ATI, and outdoors during and after ATI). This result shows that elevated levels of PM_10_ outdoors impacts the cumulative exposure to PM_10_ indoors and outdoors. Moreover, these results show that the difference in indoor and outdoor exposure was much higher during the period of ATIs (23.0 µg/m^3^) than after (6.5 µg/m^3^), which indicates that exposure to PM can be influenced by high-exposure events during specific activities and in specific microlocations during a period with persistent ATIs.

Comparing the results obtained from the AWS-B monitoring station with the PPM data shows that the cumulative outdoor exposure assessment during the period with persistent ATIs yields a similar result regardless of which method was used (57.9 µg/m^3^ for the monitoring station and 66.5 µg/m^3^ for the PPM). Outdoor exposure in the post-ATI period shows a moderately different result, where the monitoring station showed a mean value of 23.2 µg/m^3^ and the PPM 37.7 µg/m^3^. This discrepancy could be a consequence of the PPM better capturing the actual individual exposure when the participant moved throughout the city, e.g., elevated levels of PM in some areas due to the street canyon effect [40], urban green spaces and foliage [41,42], construction sites [43], or a specific action that the person was performing. Outdoor data from the PPM showed 181 instances (3% of all recordings) of PM_10_ concentrations ≥200 µg/m^3^ in the post-ATI period, while there were only 2 in the period with ATIs. The ability of the PPM to capture actual individual exposure is further illustrated by comparing indoor data from the PPM with monitoring stations, which showed lower concentrations for the PPM compared to AWS-B during the ATI period (43.5 µg/m^3^ and 57.9 µg/m^3^, respectively), and higher in the post-ATI period (31.2 µg/m^3^ for the PPM data, 23.2 µg/m^3^ for monitoring station).

**Figure 6:**
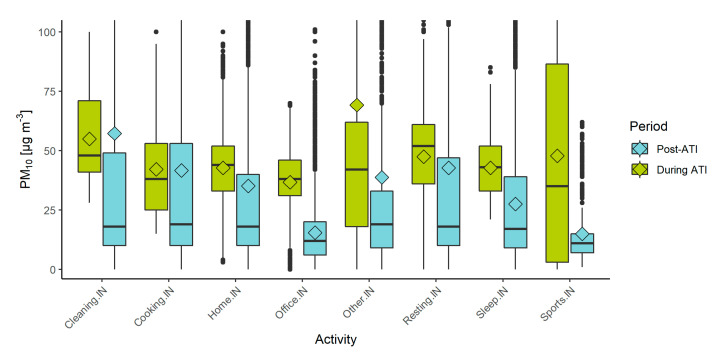
Exposure to PM_10_ for different indoor activities, during the ATI event (green) and after (blue), collected with the PPMs. Plot limited to 100 µg/m^3^ on the y axis to better illustrate the differences.

Exposure to PM_10_ (calculated using Equation (3)) while performing different indoor activities varied between 15.0 µg/m^3^ for indoor sports during the post-ATI period and 69.2 µg/m^3^ for non-determined other indoor activities during the ATI period, as shown in Figure 6. Indoor activities during the ATI period had mostly the highest mean values of PM_10_, with the exception of cleaning and cooking, which were almost the same as in the post-ATI period. The largest difference was for indoor sporting activities, which had a difference of 32.9 µg/m^3^ between the two periods. Engaging in sporting activities indoors can prompt the person in the enclosed space to open windows or doors to cool down and ventilate the room, consequently causing an influx of air with a higher concentration of PM_10_ during an ATI event [44]. The next-highest difference was for other indoor activities (69.2 µg/m^3^ during ATI and 38.7 µg/m^3^ during post-ATI period), which often included a combination of different already-listed activities and various others. This difference is more difficult to explain due to the variability of different activities, which might include dust resuspension, use of incense, having an open window, etc.

On the other hand, mean concentrations of PM_10_ for the “cleaning” activity were higher in the post-ATI period by 2.3 µg/m^3^, and only slightly lower for cooking (difference of 0.5 µg/m^3^), though the median shows much lower values. Cooking and cleaning are important sources of indoor PM, and sporadic high emission events such as frying of food or dust resuspension during cleaning can increase exposure to a higher concentration than exposure during ATI events [45,46,47]. These events are not captured by monitoring stations and can even be missed by stationary indoor sensors if they are not present in all rooms. Individual-level monitoring shows data on a very granular level and includes specific high-emission events.

**Figure 7:**
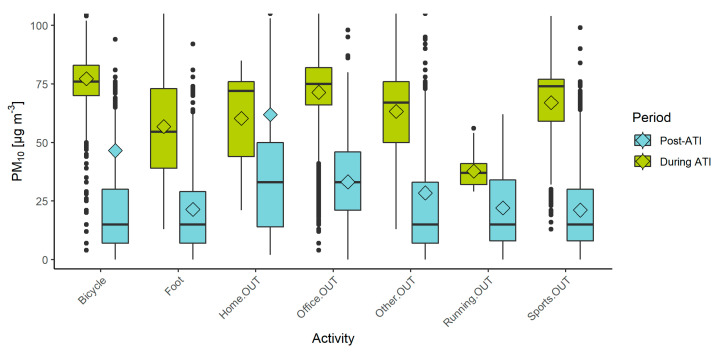
Exposure to PM_10_ for different outdoor activities, during (green) and after the period with persistent ATI (blue), collected with the PPMs. Plot limited to 100 µg/m^3^ on the y axis to better illustrate the differences between mean and median values.

Figure 7 illustrates the differences between the distribution, mean, and median values of PM_10_ for all outdoor activities during the ATI period and post-ATI. Almost all outdoor activities show higher recorded values during the ATI period than the post-ATI period. The differences range from 15.7 µg/m^3^ for running to 45.9 µg/m^3^ for outdoor sports that don’t include running, with the exception of the “home” activity, which was higher during the post-ATI period by 1.7 µg/m^3^. A possible explanation for a higher concentration would be that outdoor activities around the home can include gardening, burning of gardening and agricultural residues [48], and home-improvement activities that often include some kind of construction (sanding wood, mixing cement, demolishing objects, etc.) which present a source of particulate matter [49,50]. Such activities can be expected to occur more frequently during the post-ATI period with clearer weather and could elevate the concentrations of PM_10_. Participants could also differently interpret specific outdoor “home” activities, e.g., resting on a semi-enclosed balcony. Although the mean values do not differ much for the “Home.OUT” activity, the median values show that there is still a difference between these two time periods and indicate that the higher mean value of outdoor activities in the post-ATI period could be influenced by a few high-exposure events. For illustration, if the high-exposure events (>100 µg/m^3^) are removed, the mean value decreases from 61.9 µg/m^3^ to 30.1 µg/m^3^, while the median value drops from 33 µg/m^3^ to 27 µg/m^3^.

Riding a bicycle shows the highest recorded mean value of PM_10_ among all pre-classified activities, with 77.2 µg/m^3^. As cyclists cover large distances and areas in an urban environment compared to pedestrians, they could potentially cross through more areas with higher concentrations of PM_10_, e.g., construction areas, heavy traffic, and intersections [51], and increase their exposure based on extreme values, high-exposure events, and higher spatiotemporal variations [52]. Moreover, cyclists are regularly forced to share traffic lanes with motor vehicles, which increases their exposure to PM [53]. A risk-benefit balance assessment between active travel-related physical activity and exposure to air pollution shows that in areas with PM_2.5_ concentrations of >100 µg/m^3^, harms would exceed benefits after 90 min of bicycling per day or more than 10 h of walking per day [54]. On the other hand, our research revealed that there is a fairly large discrepancy between the mean and median value for cycling in the post-ATI period, which is a consequence of several brief high-exposure events (>100 µg/m^3^) that represent 4.3% of the recorded values for the cycling activity. If these specific events are omitted, the mean value decreases from 46.5 µg/m^3^ to 19.9 µg/m^3^, which is close to the median value of 15 µg/m^3^ (14 µg/m^3^, after high-exposure values are removed).

As evident for the “foot” (walking) activities in the post-ATI period in Figure 7, pedestrians had a smaller difference between their mean and median value of exposure of 1.4 µg/m^3^. Moreover, the walking activity had a smaller number of high-exposure events of >100 µg/m^3^ (0.9% of all recorded values), which had a lower mean value of 181.3 µg/m^3^, compared to values for cycling >100 µg/m^3^ with a mean value of 633.4 µg/m^3^. A similar trend is present for the period with ATIs. Pedestrians are exposed to varying concentrations of PM throughout the urban environment based on different types of road, traffic volume, time of day, and season [55], or specific high-exposure events, e.g., queuing by or walking across a crosswalk [56], which influence their cumulative exposure. As they move slower and cover less distance than cyclists, the variability can be lower, moreover, they are less frequently exposed to direct traffic exhaust than cyclists. Exposure of pedestrians is influenced by background concentrations and on smaller local roads by the pedestrians themselves who resuspend dust and might increase the concentrations of coarser fractions of PM [57].

These results illustrate how the difference in exposure between the ATI and the post-ATI period for the outdoor activities is larger than for the indoor activities, indicating that specific activities and the associated sources of PM increase exposure indoors.

## 4. Limitations

Certain limitations were observed in this study. Data in the ICARUS sampling campaign in Ljubljana were collected in only one non-heating season at the end of February and the beginning of March, which resulted in only a single period with persistent ATIs. This research could be further improved by analyzing multiple periods in different years and in different locations/cities, though this would present the logistical challenge of organizing yearly sampling campaigns with hundreds of participants. The sampling campaign began just as the period of persistent ATIs started, which prevented any comparisons with data prior to the ATI period.

Personal monitors based on low-cost sensors often have issues regarding their usability, data accuracy, and technical malfunctions. The PPMs used in this study frequently stopped working, did not record data, had poor accuracy of GPS data, ran out of battery, and showed data that were clearly erroneous, which resulted in data loss and also increased the workload of researchers and field workers. PM values recorded for certain activities, e.g., running, could be erroneous due to the aforementioned issues.

An additional limitation of the study was the manual logging of activity data by the participants, who frequently logged data for several days at once and sometimes mistakenly chose the wrong activity, which increased the possibility of errors. The activities did not have the same frequency in the two periods (during and post-ATI), and some were recorded only in one period and consequently eliminated from this analysis.

## 5. Conclusions

Within the scope of this research, an analysis of the applicability of personal PM monitor-based individual-level exposure assessments for capturing the spatiotemporal variability of individual exposure profiles was made. Two contrasting periods in terms of meteorological conditions and air quality—a period with persistent atmospheric thermal inversions (ATIs) and a post-ATI period—were used to determine how the aforementioned approach can assess exposure during specific activities and in specific microlocations. Data were collected on indoor and outdoor activities performed by participants in Ljubljana. Exposure was compared by observing the statistical values of the recorded data in the two distinct periods and comparing it with data collected from monitoring stations.

Results showed that the difference in indoor and outdoor exposure was much higher during the period of ATIs (23.0 µg/m^3^) than after (6.5 µg/m^3^). Indoor activities generally showed less difference in mean and median values, with cooking and cleaning having higher values in the post-ATI period than during the ATI. On the other hand, almost all outdoor activities had higher PM values during the ATI than after. Several conclusions can be drawn from these results:

1. Periods with persistent ATIs present a fitting opportunity to assess the applicability of personal monitors to capture the spatiotemporal variability of indoor and outdoor exposure. A clear distinction in terms of PM concentrations between the two periods provides an opportunity to observe how high-exposure events can influence cumulative exposure;
2. Exposure to PM_10_ is higher during periods with persistent ATIs, when ambient concentrations increase due to specific meteorological conditions. This is evident indoors and outdoors and for almost all activities, except for a few that are mainly influenced by the PM_10_ associated with the respective activity. Indoor concentrations are lower than the outdoor concentrations during the period with ATIs, though they are still higher than indoor and outdoor concentrations in the post-ATI period;
3. Using activity data enables an individual-level scale analysis of exposure and illustrates that the influence of activities on exposure indoors should not be disregarded when assessing cumulative exposure. Activities can directly, e.g., cooking and cleaning, or indirectly reduce air quality, e.g., opening a window during a period with poor outdoor air quality;
4. Measuring exposure on an individual level is necessary to capture high-exposure events in microlocations. These results showed that several high-exposure events can greatly raise exposure levels. Additionally, personal monitors can detect trends and show how specific routines influence exposure;
5. These measurements confirm that there are high levels of exposure indoors even in high-income countries that mostly don’t use solid fuels for cooking and heating. A better understanding of activity-specific exposure could provide a basis for policies that can more accurately address exposure to poor air quality.

Overall, this study demonstrated that utilizing personal monitors in exposure assessments can provide better spatiotemporal resolution and capture specific high-exposure events. These devices provide an ancillary tool that can indicate trends and guide further research.

Future work should include more detailed activities and a better spatiotemporal resolution. Personal monitors could be further improved to better record, store, harmonize and transfer data, detect outliers, have on-the-fly calibration options, and integrate multiple devices. Reducing the proportion of data that are recorded by human input via an approach with automated activity recognition could improve exposure assessments [58]. Exposure models that rely solely on outdoor measuring stations or indoor stationary devices fail to capture high-exposure events and could be improved by integrating data from personal monitors. Moreover, data from personal monitors could be integrated into agent-based models to supplement other data sources [59], e.g., monitoring stations, statistical and demographic data, etc.

This research addressed exposure to particulate matter, though there are numerous other air pollutants that could be further investigated by employing personal monitors. Moreover, current AQ guidelines often do not include indoor environments or individual-level exposure. Results obtained in the scope of this research should be further developed and transferred into policy, to include approaches that utilize data on a personal scale and the specifics related to human behavior in urban environments.

## Figures and Tables

**Table 1:** Automatic weather stations (AWS) used to determine ATIs, their locations, elevations, and parameters collected.

Station	Meters above Sea Level	Coordinates	Parameters
AWS-B	299 m	46.0654 N, 14.5123 E	Temperature, PM_10_
AWS-T	692 m	46.0940 N, 14.3713 E	Temperature
AWS-K	1742 m	46.2978 N, 14.5335 E	Temperature

**Table 2:** Number of instances for each indoor activity during the ATI period and in the post-ATI period.

Period	Cleaning	Cooking	Home	Office	Other	Resting	Sleeping	Sports
ATI	395	472	8535	1319	632	1414	3638	142
post-ATI	2060	3686	41,320	11,346	4921	15,525	19,287	373

**Table 3:** Number of instances for each outdoor activity during the ATI period and in the post-ATI period.

Period	Bicycle	Foot	Home	Office	Other	Running	Sports
ATI	1057	538	184	1170	366	61	295
post-ATI	1312	1822	1583	967	3479	294	1825

## Data Availability

Data available on request due to privacy restrictions.
